# Analysis of Pediatric Pulpotomy, Pulpectomy, and Extractions in Primary Teeth Revealed No Significant Association with Subsequent Root Canal Therapy and Extractions in Permanent Teeth: A Retrospective Study

**DOI:** 10.3390/pediatric16020038

**Published:** 2024-05-31

**Authors:** Arash Farhadian, Mayce Arreem Issa, Karl Kingsley, Victoria Sullivan

**Affiliations:** 1 Department of Advanced Education in Pediatric Dentistry, School of Dental Medicine, University of Nevada, Las Vegas, 1700 West Charleston Blvd, Las Vegas, NV 89106, USAvictoria.sullivan@unlv.edu (V.S.); 2 Department of Clinical Sciences, School of Dental Medicine, University of Nevada, Las Vegas, 1700 West Charleston Blvd, Las Vegas, NV 89106, USA; 3 Department of Biomedical Sciences, School of Dental Medicine, University of Nevada, Las Vegas, 1001 Shadow Lane, Las Vegas, NV 89106, USA

**Keywords:** pulpotomy, pulpectomy, endodontic, root canal therapy, pediatric prevalence, risk factors

## Abstract

Recent evidence suggests that an ever-growing number of pediatric patients require invasive treatments such as root canal therapy (RCT) in their permanent dentition, albeit with little information about risk factors such as prior invasive treatments of pulpotomy or pulpectomy in their primary dentition. Therefore, the primary objectives of this study were to determine the number of pediatric patients who have had any type of invasive treatment in their primary teeth, to assess their association with any subsequent invasive treatment (root canal therapy, extractions) in their permanent dentition, and to assess these trends over time. This retrospective study utilized summary data from a clinical pediatric patient pool (ages 0–17) over the period of 2013–2022. This analysis revealed that pediatric patients requiring pulpotomies and pulpectomies in primary dentition declined between 2013 (*n* = 417, *n* = 156) and 2022 (*n* = 250, *n* = 12), while root canal therapy (RCT) in permanent dentition increased six-fold from *n* = 54 to *n* = 330. In addition, few (7.8%) patients with RCT had a previous history of pulpotomy or pulpectomy, which suggests that invasive treatments performed in primary dentition have no direct association with the subsequent need for invasive treatments in permanent dentition, although more research is needed to determine the explanations for these observations.

## 1. Introduction

Many modern integrative technologies, such as Artificial Intelligence (AI), are now being used to predict diagnosis and improve treatment planning for dental patients [1,2]. These technologies rely on deep learning (DL) and machine learning (ML), which allow computers to create patterns and determine associations between current or past dental conditions and treatments with future dental needs and subsequent diagnoses [3,4,5]. New evidence has demonstrated numerous examples of applications and algorithms (based on AI, DL or ML) to improve detection, diagnosis and treatment planning for dental diseases, such as dental caries [6,7].

However, many researchers have suggested that these developments in future prediction models for diagnosis and treatment planning will need to rely on epidemiologic studies of prevalence and association in order to successfully predict these future outcomes [8,9]. For example, systematic reviews of AI applications for use in diagnosing and treating endodontic infections—particularly in geographic areas that lack a specialist to treat or review these cases—have demonstrated high levels of accuracy and precision using these models [10,11]. However, most of these studies were specifically focused on using methods and applications to review radiographs for endodontic diagnosis and treatment planning—with little to no evidence that these models have been trained in future prediction and diagnosis [12,13].

However, recent evidence suggests that an ever-growing number of pediatric patients are requiring the invasive treatment of root canal therapy (RCT) in their permanent dentition, albeit with little information about risk factors such as prior invasive treatments of pulpotomy or pulpectomy in their primary dentition [14,15]. Although some studies have reviewed alternative factors, such as dental trauma or periapical status, and the need for subsequent endodontic treatment, few (if any) studies to date have evaluated the potential associations between pediatric deciduous vital pulp therapy or pulpotomy and pulpectomy and the subsequent need for root canal treatment in permanent teeth among pediatric patients [16,17]. Strong evidence regarding the efficacy and indications for pediatric vital pulp therapy, as well as partial or full pulpotomy, has been disseminated, although it does not provide any indication regarding the prevalence or incidence of subsequent treatments on different teeth within those same individuals [18,19,20].

Although some models have recently been developed using the primary caries experience to predict future permanent teeth caries among pediatric patients, no studies to date have evaluated the association between pulpotomy or pulpectomy among primary dentition and future endodontic treatment in permanent teeth within those same individuals [21]. Due to the lack of available evidence in this area and to take a more holistic view by including all types of treatments, the primary objective of this study was to assess and evaluate the prevalence of pediatric patients who have had invasive treatments of pulpotomy, pulpectomy, or extractions in primary teeth, and then to assess their association with subsequent invasive treatments of RCT or extractions in permanent teeth.

## 2. Materials and Methods

### 2.1. Study Review and Approval

This study involved a retrospective analysis of summarized patient data and was therefore deemed Exempt under Federal Regulation 45 CFR 46. In brief, this regulation covers the study of existing data, documents or records that currently exist and are not prospectively collected, where (1) participants cannot be directly identified; and (2) participants cannot be identified through identifiers linked to them. Based upon this information, the Institutional Review Board (IRB), under the Office for the Protection of Research Subjects (OPRS) at the University of Nevada, Las Vegas (UNLV), approved this study under Protocol 1619329-1, which was titled “Retrospective Analysis of Oral Health Status of Dental Population” as Research Exempt.

This study consisted of the review and subsequent analysis of previously collected, non-identifiable pediatric data and information from the UNLV School of Dental Medicine (SDM) clinic. Based upon this background information, Informed Consent was not needed and was waived pursuant to the Basic Health and Human Services (HHS) Policy for the Protection of Human Research Subjects (46.101), specifically regarding the IRB exemption for Exempt research not involving patient contact or prospective patient data collection. Other than basic demographic information, such as age, race or ethnicity, and sex, the study authors had no patient identification or other patient-specific information provided.

### 2.2. Data Analysis

The basic demographic information, including the age of the patient at the time of dental treatment or service, as well as the patient’s sex or gender, and race or ethnicity, was provided. The patient treatment and billing requirements mandate completed patient information (sex, age, treatment or procedure code, date of service); therefore, no data points were excluded, although some patient records do not contain race or ethnicity information, which is voluntary. More detailed information regarding the dental services and treatments was obtained for these patients, which included therapeutic pulpotomy, pulpectomy, endodontic therapy—molar, endodontic therapy—premolar, endodontic therapy—anterior tooth, extraction—erupted tooth or exposed root, and surgical removal of erupted tooth. Summary data, including the date of service, sex, age of patient, and race or ethnicity, were imported into Microsoft Excel 2021, Office 365 Version from Microsoft (Redmond, WA, USA). Descriptive statistics were generated, which included the overall summary and associated percentages that were calculated from the retrospective information derived from the clinic data. In addition, Pearson’s correlation (R) was calculated to evaluate the direction and magnitude of any observed changes over the study time period (2013 to 2022), which also allowed for the calculation of the coefficient of determination (R^2^).

Finally, to determine the association between primary tooth pulpotomy, pulpectomy, or extraction and subsequent permanent tooth root canal therapy (RCT) or extraction, the odds ratio (OR) and relative risk (RR) were also calculated, as previously described [22], using GraphPad Prism 9 software (Boston, MA, USA). The relative risk describes the risk of an event (e.g., permanent tooth RCT or extraction) in one group (those with previous primary tooth pulpotomy, pulpectomy, or extraction) versus the risk of the event in another group (those without previous primary tooth pulpotomy, pulpectomy, or extraction). For the RR, 1 is normal risk, >1 is elevated risk, and <1 is lower than normal risk. The odds ratio describes the likelihood or odds of an event in any given group, such as the odds of a permanent tooth RCT or extraction in the group with previous primary tooth pulpotomy, pulpectomy, or extraction. Similar to the RR, the OR of 1 is normal odds, >1 is greater odds, and <1 is lower odds. For all the calculations performed, the 95% confidence interval (CI) and *p*-value are provided.

## 3. Results

Summary data regarding the pediatric clinic patient population were retrieved and analyzed (Table 1). These data demonstrated that the pediatric clinic was nearly equally divided between females (52.2%) and males (47.8%), which was not significantly different from the overall patient population (52.8%, 47.2%) at this public institution within the dental clinic, *p* = 0.8412. The percentage of non-minority (22.8%) and minority (77.2%) pediatric patients was also similar to the overall clinic patient population (25.6%, 74.4%), *p* = 0.4940. A detailed breakdown of these data revealed that most of the minority patients (77.2%) in the pediatric clinic population self-identified as Latino or Hispanic (59.4%), with a smaller percentage of Black or African Americans (13.2%) and Asian or Pacific Islanders (3.4%), which was also similar to the overall clinic population (55.2%, 13.4%, 3.8%, respectively).

**Figure 1:**
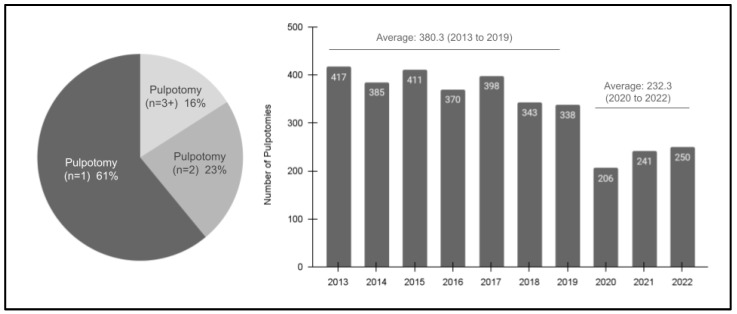
Analysis of the study sample pulpotomies (2013–2022). The majority of patients had a single pulpotomy, *n* = 1 (61%), with fewer having two, *n* = 2 (23%), or more, *n* = 3 + (16%). In addition, the annual number of pulpotomies decreased over the study period from *n* = 417 in 2013 to *n* = 250 in 2022. The average number of pulpotomies in the pre-pandemic years (2013 to 2019) was 380.3, which dropped to an average of 232.3 from 2020 to 2022.

Analysis of the pulpotomy study sample from 2013 to 2022 revealed several additional characteristics (Figure 1). Specifically, these data demonstrated that the majority of patients with pulpotomy only had one single procedure (*n* = 1; 61%), with many fewer having two (*n* = 2; 23%) or more (*n* = 3 or more; 16%). In addition, these data also demonstrate an overall declining trend in the number of procedures completed between 2013 (*n* = 417) and 2022 (*n* = 250), R = −0.882 or R^2^ = 0.778. More specifically, the average number of procedures performed during the pre-pandemic (SARS-CoV-2) years of 2013 to 2019 was 380.3, which was significantly higher than the average of 232.2 between 2020 and 2022.

**Figure 2:**
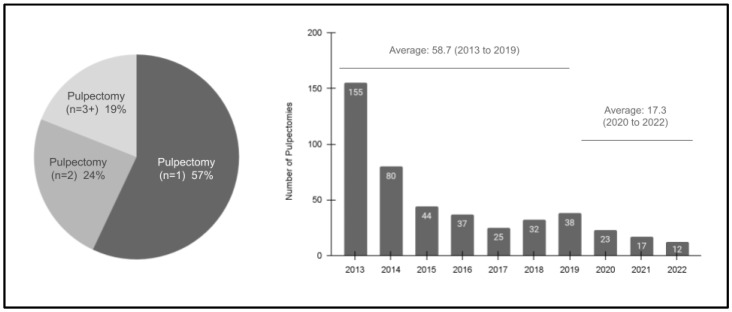
Analysis of the study sample pulpectomies (2013–2022). Most pediatric patients had a single pulpectomy, *n* = 1 (57%), with fewer having two, *n* = 2 (24%), or more, *n* = 3 + (19%), procedures. Furthermore, the annual number of pulpectomies decreased significantly over the study period from *n* = 155 in 2013 to *n* = 12 in 2022. The average number of pulpotomies in the pre-pandemic years (2013 to 2019) was *n* = 58.7, which dropped to an average of *n* = 17.3 from 2020 to 2022.

Further analysis of the pulpectomy study sample from 2013 to 2022 revealed additional information (Figure 2). In brief, these data demonstrated that the majority of pulpectomy patients also only had one single procedure (*n* = 1; 57%), with approximately one-quarter having two (*n* = 2; 24%) and with fewer needing more procedures (*n* = 3 or more; 19%). Furthermore, these data also revealed a steep declining trend in the number of procedures completed between 2013 (*n* = 155) and 2022 (*n* = 12), R = −0.784 or R^2^ = 0.616. More specifically, the average number of procedures performed during the pre-pandemic (SARS-CoV-2) years of 2013 to 2019 was 58.7, which was higher than the average between 2020 and 2022 of 17.3, although most of the declines were observed between 2013 (*n* = 155) and 2015 (*n* = 44).

**Figure 3:**
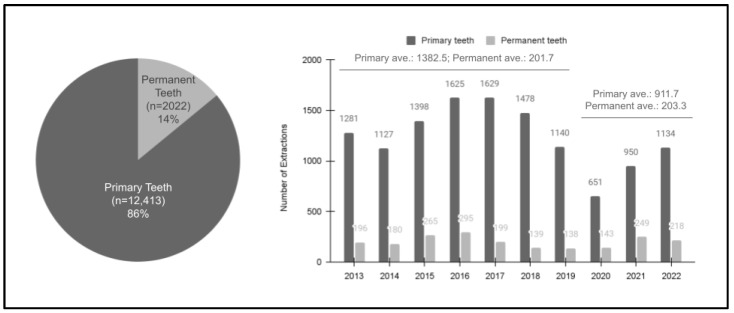
Analysis of the study sample extractions (2013–2022). Most extractions among pediatric patients were of primary teeth, *n* = 12,413 (86%), with fewer among permanent teeth, *n* = 2022 (14%). In addition, the annual number of primary extractions decreased over the study period from the pre-pandemic years (2013 to 2019, average 1382.5), which dropped to an average of 911.7 from 2020 to 2022 (R = −0.472 or R^2^ = 0.223). The extractions among permanent teeth were relatively stable over this period, ranging from an average of 201.7 (2013 to 2019) to an average of 203.3 (2020 to 2022), R = −0.153 or R^2^ = 0.024.

A question arises as to whether these declines in the prevalence of pediatric pulpotomy and pulpectomy are the results of overall declines in oral health conditions requiring more invasive treatments of primary teeth extractions instead of pulp therapy, or if the declines are the result of the dental provider’s decision to pursue extraction over pulpectomy due to either poor behavior or an attempt at a more predictable outcome that might lead to less emergency visits and parental frustration. For this reason, an analysis of extractions was also conducted (Figure 3). These data revealed that the overwhelming majority of extractions were of primary teeth (*n* = 12,413 or 86%) compared with permanent teeth (*n* = 2002 or 14%). Although there was a moderate decline in primary tooth extractions over the length of the study sample (R = −0.472 or R^2^ = 0.223), most of the declines were observed in the post-pandemic period (average 911.7 from 2020 to 2022) compared with the pre-pandemic period (average 1382.5 between 2013 and 2019). In contrast, the trend was relatively stable among the permanent teeth (R = −0.153 or R^2^ = 0.024), with similar levels in the pre-pandemic period (average *n* = 201.7 between 2013 and 2019) and post-pandemic years (average *n* = 203.3 from 2020 to 2022).

**Figure 4:**
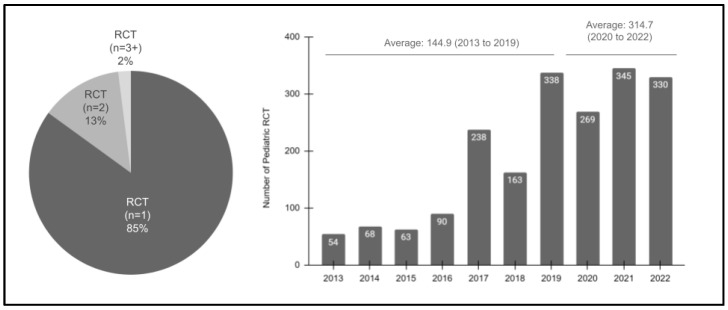
Analysis of the study sample RCT procedures (2013–2022). The majority of pediatric patients had only one RCT (85%), with fewer having two (*n* = 2; 13%) or more (*n* = 3 plus; 2%) over the study period. In addition, a strong positive increase in procedures was also observed between 2013 (*n* = 54) and 2022 (*n* = 330), R = 0.921 or R^2^ = 0.848, with the pre-pandemic yearly average (*n* = 144.9 from 2013 to 2019) increasing up to the post-pandemic yearly average (*n* = 314.7).

Continued analysis of these data then revealed that the overwhelming majority (85%) of pediatric patients had a single RCT procedure (Figure 4). More specifically, only a small percentage of patients had two (13%) or more (2%) RCT procedures during the evaluated study period. In addition, a strong positive increase in procedures was also observed between 2013 (*n* = 54) and 2022 (*n* = 330), which was significant, R = 0.921 or R^2^ = 0.848. The differences were evident between the pre-pandemic yearly average (*n* = 144.9 from 2013 to 2019) and the post-pandemic yearly average (*n* = 314.7).

To compare and evaluate potential confounding variables, the demographic characteristics of pulpotomy, pulpectomy, and root canal therapy (RCT) patients among the pediatric study sample were evaluated (Table 2). This analysis revealed that nearly equal percentages of females and males had pulpotomies (50.3% and 49.7%, *p* = 0.6889), pulpectomies (46.1% and 53.9%, *p* = 0.3169), extractions (49.8% and 50.2%, *p* = 0.6889) and RCT (52.2% and 47.8%, *p* = 0.6889), which was not statistically different from the overall clinic population sample.

However, a significantly larger percentage of patients undergoing any of these procedures (pulpotomy, pulpectomy, extraction, or RCT) was from a minority (non-White) compared with the overall pediatric clinic population. More specifically, the percentages of minority and non-minority patients undergoing pulpotomies (96.8% and 3.2%, *p* = 0.0001), pulpectomies (87.5% and 12.5%, *p* = 0.0090), extractions (95.4% and 4.6%, *p* = 0.0001), and RCT (93.3% and 6.7%, *p* = 0.0001) were significantly different from the overall pediatric population (77.2% and 22.8%, respectively). Most of the patients undergoing these procedures were Latino or Hispanic, with the remainder identified as Black or African American, Asian or Pacific Islander or Other.

**Figure 5:**
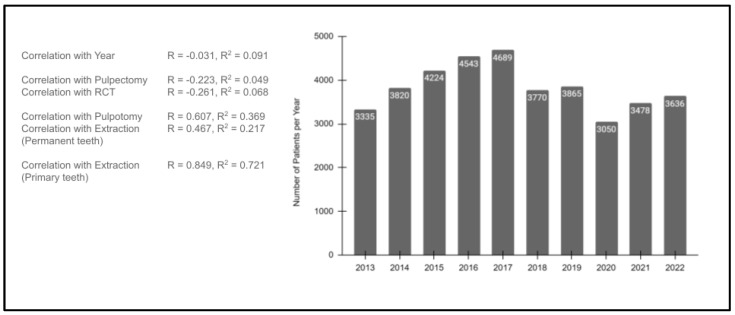
Analysis of the study sample patients seen per year (2013–2022). The overall number of pediatric patients seen each year was relatively stable (2013, *n* = 3335; 2022, *n* = 36,356), R = −0.301 and R^2^ = 0.091. Low correlations were observed between the number of patients seen and pulpectomy (R = −0.223, R^2^ = 0.049) or RCT (R = −0.261, R^2^ = 0.068). Moderate associations were found between number of patients seen and pulpotomies (R = 0.607, R^2^ = 0.369) or extractions of permanent teeth (R = 0.467, R^2^ = 0.217). More significant associations were found between extractions of primary teeth and the number of patients seen (R = 0.849, R^2^ = 0.721).

To evaluate the potential impact of patient visits in both the pre- and post-pandemic years, an analysis of the total number of patients seen in each year was performed (Figure 5). These data revealed the overall number of pediatric patients seen each year was relatively stable over time, with *n* = 3335 patients seen in 2013 and *n* = 36,356. Although there were some fluctuations over the time period evaluated, the overall linear correlation was R = −0.301, with the R^2^ = 0.091, which indicates a very low association between any change in the number of patients seen over the overall study period.

In addition, the correlations between the total number of patients seen and each of the analyzed procedures were assessed. This analysis revealed very low correlations between the number of patients seen and either pulpectomy (R = −0.223, R^2^ = 0.049) or RCT (R = −0.261, R^2^ = 0.068). More moderate associations were found between pulpotomies (R = 0.607, R^2^ = 0.369) and extractions of permanent teeth (R = 0.467, R^2^ = 0.217). However, a more significant association was found between extractions of primary teeth and the number of patients seen (R = 0.849, R^2^ = 0.721).

**Figure 6:**
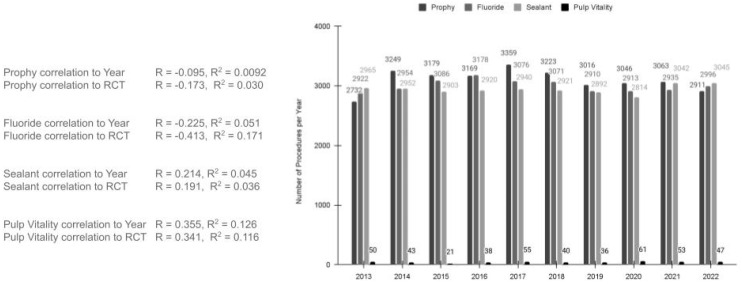
Analysis of the dental prophylaxis, fluoride treatments, and dental sealants (2013 to 2022). The number of dental prophy procedures were consistent between 2013 (*n* = 2732) and 2022 (*n* = 2911), R = −0.095 and R^2^ = 0.0092, with no significant association between dental prophylaxis and RCT (R = −0.173 and R^2^ = 0.030). Fluoride treatments were also consistent between 2013 (*n* = 2922) and 2022 (*n* = 2996), R = −0.225 and R^2^ = 0.051 and had no significant correlations with RCT (R = −0.413 and R^2^ = 0.171). The number of dental sealants placed was also found to be relatively consistent between 2013 (*n* = 2965) and 2022 (*n* = 3045), R = 0.214 and R^2^ = 0.046 and was not significantly correlated with RCT, R = 0.190, R^2^ = 0.036. Similarly, no significant changes in pulp vitality testing were found between 2013 and 2022 (*n* = 50 and *n* = 47, respectively), R = 0.355 and R^2^ = 0.126, which were not significantly correlated with RCT, R = 0.341, R^2^ = 0.116.

An analysis to evaluate any potential influence from other dental procedures, such as dental prophylaxis (cleaning teeth for the removal of plaque and tartar buildup), fluoride varnish or treatments, and dental sealants, was also performed (Figure 6). This analysis demonstrated a nearly consistent level of dental prophy procedures each year between 2013 (*n* = 2732) and 2022 (*n* = 2911), R = −0.095 and R^2^ = 0.0092. Further analysis also revealed no significant association between dental prophylaxis and RCT (R = −0.173 and R^2^ = 0.030). Analysis of fluoride treatments also revealed few fluctuations in the number of annual procedures completed between 2013 (*n* = 2922) and 2022 (*n* = 2996), R = −0.225 and R^2^ = 0.051, which were also not found to have significant correlations with RCT (R = −0.413 and R^2^ = 0.171). Similarly, the number of dental sealants placed was also found to be relatively consistent between 2013 (*n* = 2965) and 2022 (*n* = 3045), R = 0.214 and R^2^ = 0.046, and it was not significantly correlated with RCT, R = 0.190, R^2^ = 0.036. Finally, to determine whether alternative procedures, such as indirect pulp capping or vital pulp therapy, may have influenced these findings, the pulp vitality tests that are required before proceeding to these procedures were evaluated. This analysis revealed no significant changes in pulp vitality testing between 2013 and 2022 (*n* = 50 and *n* = 47, respectively), R = 0.355 and R^2^ = 0.126, and it was not significantly correlated with RCT, R = 0.341, R^2^ = 0.116.

Finally, to evaluate the risk associated with pulpotomy or pulpectomy and subsequent RCT among pediatric patients, the odds ratio (OR) and relative risk (RR) were calculated (Table 3). For those patients with a history of pulpotomy or pulpectomy, the relative risk (RR) compared with those without a history of pulpotomy or pulpectomy for subsequent RCT therapy was low (RR = 0.2360), *p* < 0.0001. Similarly, the odds ratio for having RCT if the patient had already had a pulpotomy or pulpectomy was also very low (OR = 0.2193), *p* < 0.0001.

## 4. Discussion

The primary goal of this study was to analyze pediatric patients who have had invasive treatments of primary tooth pulpotomies, pulpectomies, and extractions and determine their relationship with subsequent RCT and extractions within permanent teeth. This study clearly demonstrated several temporal changes, such as the decrease in pulpotomies and pulpectomies and the corresponding increase in root canal therapies over the same time interval. However, further analysis demonstrated that these were, in fact, not the same patients and that a previous history of pulpotomy or pulpectomy was not a predictor of future endodontic therapy within this large clinic population.

In fact, these data may be among the first to describe these inverse temporal observations, although other studies have evaluated other relationships, such as the associations between pain and the efficacy of pulpotomy versus RCT for these patients [23,24,25]. In addition, many studies have evaluated various biomaterials, such as mineral trioxide aggregate (MTA) or formocresol, to predict successful outcomes of endodontic treatments, although these studies did not evaluate the initial risk factors that may have led to the increased probability of needing these procedures [26,27]. In fact, most of the predictive models developed for endodontics focus on either the diagnostic and treatment planning aspects or models for the prediction of intraoperative pain [11,12,13,28,29,30].

Although other studies have found the relationship between previous pediatric dental outcomes in primary teeth, such as caries experience, to be a strong predictor of future dental outcomes in permanent teeth, this study did not find a similar pattern between endodontic treatment of primary teeth and endodontic treatment of permanent teeth [6,7,8,9]. Some research has suggested a potential explanation for this may be due to the preference and prevalence of dental extractions, which many dental and oral healthcare providers have suggested may be an effective and cost-saving method for dealing with invasive and complicated procedures involving primary teeth [31,32]. However, the data from this study strongly suggest that extractions (both among primary and permanent teeth) did not change during the same time period that experienced decreases in the prevalence of pulpotomies and pulpectomies.

This may be due, in part, to better adherence to the American Academy of Pediatric Dentistry (AAPD)’s recommendations for alternative and less invasive forms of treatment, such as indirect pulp therapy (IPT) due to its higher success rates than pulp therapy in primary teeth where pulp tissue may not be directly manipulated but a biocompatible material prevents microleakage and facilitates remineralization of dentine [33,34,35]. These observations may also have been influenced, in part, by the changes in dental utilization and service barriers during the SARS-CoV-2 (COVID-19) pandemic, which decreased access and increased obstacles to care for many pediatric patients, particularly those with socioeconomic challenges [36,37,38]. However, the findings that long-term pediatric patients experiencing RCT, both before and after the onset of the pandemic, had limited experience with endodontic treatments such as pulpotomy or pulpectomy suggest that alternative factors may be at work.

In order for future studies to account for these variations, it is important to discuss the limitations of this study. First, this is a retrospective study of previously collected data and may have bias intrinsic to the study sample due to the nature of this low-income, predominantly minority clinic population, which may face significant challenges and barriers to health care access and oral health prevention [22,39]. In addition, the reduced nature of some of the procedures performed during the COVID-19 pandemic may have been due to changes in protocols for dental care, which reduced the number of available openings for treatment, as well as changes in patient or parent behaviors, including delays in care due to safety concerns [40,41]. Finally, additional variables that were not the focus of this retrospective investigation of the associations between pulpotomy, pulpectomy and RCT, such as alternative procedures that include pulp capping or vital pulp therapy, may be indirect confounding variables and can be the subject of future investigations.

For example, some of the declines in the prevalence of pulpotomies and pulpectomies may be the results of recent systematic reviews that inform evidence-based practice, which now favor attempts at less invasive procedures, such as indirect pulp therapy and incomplete caries excavation [42,43]. Additionally, pulpotomies and pulpectomies are more time-consuming and involve procedures that may not be as well tolerated by children with behavioral problems [44,45]. With the increasing prevalence of dental anxiety and behavioral problems noted among children in recent years, some clinical providers may be avoiding pulp exposure more often when a less invasive approach could provide a potential alternative [46,47].

Furthermore, some of the increases in RCT therapy may be due to the possibility that some pediatric patients may be referred to the clinic solely for RCT, which may have more limited opportunities to be facilitated in the local dental provider network, which may lack sufficient specialists to perform these procedures [48,49]. In addition, many other local dental providers no longer accept Medicaid and other forms of public assistance, which may increase the growing number of families and young children depending upon this type of coverage to seek this type of care at this public dental school clinic [50,51]. However, future research that can evaluate these factors in more detail will be needed to adequately explore whether any of these confounding factors are responsible for the observations revealed in this study.

## 5. Conclusions

This study evaluated pediatric patients who had endodontic therapy in primary teeth, such as pulpotomies and pulpectomies, and found no clear association with subsequent endodontic therapy in permanent dentition. This analysis also revealed that the relative risk and odds ratio for endodontic therapy in permanent teeth with a previous history of either pulpotomy or pulpectomy procedures was very low. These observations suggest that there may be factors other than the history of pulpotomy and pulpectomy that may influence the need for RCT in permanent teeth, revealing the need for further research into the additional risk factors and other oral health conditions that might increase the likelihood or probability for these procedures among this patient population [52]. Dissemination of evidence-based public health information and training for parents and children regarding proper oral health and good oral hygiene practices may address the challenges of reduced health literacy and may also have the potential to reduce the need for these types of invasive procedures.

## Figures and Tables

**Table 1:** Demographic analysis of the pediatric clinic population.

Demographics	Pediatric Clinic Population (*n* = 24,460)	UNLV-SDM Patient Clinic Population	Statistical Analysis
*Sex*			X^2^ = 0.040, d.f. = 1 *p* = 0.8412
Females (*n* = 12,758)	52.2%	52.8%	
Males (*n* = 11,702)	47.8%	47.2%	
*Ethnicity or Race*			X^2^ = 0.468, d.f. = 1 *p* = 0.4940
Caucasian or White (Non-Minority)	22.8%	25.6%	
Minority (Non-White)	77.2%	74.4%	
Detailed analysis of Minorities			
(Latino or Hispanic)	(59.4%)	(55.2%)	
(Black or African American)	(13.2%)	(13.4%)	
(Asian or Pacific Islander)	(3.4%)	(3.8%)	
(Other)	(1.2%)	(2.0%)	

**Table 2:** Demographic analysis of patients with procedures within the study sample.

	Pulpotomy Study Sample 2013–2022 (*n* = 3359)	Pulpectomy Study Sample 2013–2022 (*n* = 463)	Extractions within Study Sample 2013–2022 (*n* = 14,435)	RCT within Study Sample 2013–2022 (*n* = 1958)
Sex				
Females	50.3%	46.1%	49.8%	50.3%
Males	49.7%	53.9%	50.2%	49.7%
Statistical Analysis	X^2^ = 0.160, d.f. = 1 *p* = 0.6889	X^2^ = 1.002, d.f. = 1 *p* = 0.3169	X^2^ = 0.160, d.f. = 1 *p* = 0.6889	X^2^ = 0.160, d.f. = 1 *p* = 0.6889
Race/Ethnicity				
Caucasian or White (Non-Minority)	3.2%	12.5%	4.6%	6.7%
Minority (Non-White)	96.8%	87.5%	95.4%	93.3%
Statistical Analysis	X^2^ = 22.586, d.f. = 1 *p* = 0.0001	X^2^ = 6.832, d.f. = 1 *p* = 0.0090	X^2^ = 18.295, d.f. = 1 *p* = 0.0001	X^2^ = 14.455, d.f. = 1 *p* = 0.0001
Detailed Analysis of Minorities				
(Latino or Hispanic)	(87.3%)	(65.6%)	(86.9%)	(79.8%)
(Black or African American)	(6.5%)	(13.6%)	(5.0%)	(10.6%)
(Asian or Pacific Islander)	(1.1%)	(3.9%)	(2.4%)	(1.9%)
(Other)	(1.9%)	(4.4%)	(1.1%)	(1.0%)

**Table 3:** Analysis of the relative risk (RR) and odds ratio (OR) for RCT among study samples.

	Exposed Group (Previous History of Pulpotomy/Pulpectomy) *n* = 3882	Non-Exposed Group (No Previous Pulpotomy/Pulpectomy) *n* = 20,638	Statistical Analysis
Events (RCT)	RCT with previous history of pulpotomy or pulpectomy *n* = 82	RCT with no previous history of pulpotomy or pulpectomy *n* = 1876	Relative risk RR = 0.2360 95% CI [0.1897, 0.2936] *p* < 0.0001
Non-events (RCT)	No RCT among those with previous history of pulpotomy or pulpectomy *n* = 3740	No RCT among those with no previous history of pulpotomy or pulpectomy *n* = 18,762	Odds ratio OR = 0.2193 95% CI [0.175, 0.274] *p* < 0.0001

Key: RR = relative risk (1 is normal risk, >1 is elevated risk, <1 is lower than normal risk); OR = odds ratio (1 is normal odds, >1 is greater odds, <1 is lower odds); CI = confidence interval.

## Data Availability

The primary data may be available upon request from the corresponding author. These data are not publicly available according to the protection parameters for the study protocol, which were required by the IRB and OPRS for the study approval.

## Abbreviations

Root canal therapy (RCT), Artificial Intelligence (AI), deep learning (DL), machine learning (ML), Institutional Review Board (IRB), Office for the Protection of Research Subjects (OPRS), University of Nevada, Las Vegas (UNLV), School of Dental Medicine (SDM), Health and Human Services (HHS), Pearson’s correlation (R), coefficient of determination (R2), odds ratio (OR), relative risk (RR), confidence interval (CI), severe acute respiratory syndrome (SARS-CoV-2), mineral trioxide aggregate (MTA), American Academy of Pediatric Dentistry (AAPD), indirect pulp therapy (IPT).
